# Germline loss-of-function variants in the *BARD1* gene are associated with early-onset familial breast cancer but not ovarian cancer

**DOI:** 10.1186/s13058-019-1137-9

**Published:** 2019-04-29

**Authors:** Nana Weber-Lassalle, Julika Borde, Konstantin Weber-Lassalle, Judit Horváth, Dieter Niederacher, Norbert Arnold, Silke Kaulfuß, Corinna Ernst, Victoria G. Paul, Ellen Honisch, Kristina Klaschik, Alexander E. Volk, Christian Kubisch, Steffen Rapp, Nadine Lichey, Janine Altmüller, Louisa Lepkes, Esther Pohl-Rescigno, Holger Thiele, Peter Nürnberg, Mirjam Larsen, Lisa Richters, Kerstin Rhiem, Barbara Wappenschmidt, Christoph Engel, Alfons Meindl, Rita K. Schmutzler, Eric Hahnen, Jan Hauke

**Affiliations:** 1 Center for Hereditary Breast and Ovarian Cancer, Center for Integrated Oncology (CIO), University of Cologne, Faculty of Medicine and University Hospital Cologne, Kerpener Str. 34, 50931 Cologne, Germany; 2 Institute for Human Genetics, University Hospital Muenster, Muenster, Germany; 3 Department of Gynaecology and Obstetrics, University Hospital Duesseldorf, Heinrich-Heine University Duesseldorf, Duesseldorf, Germany; 4 Institute of Clinical Molecular Biology, Department of Gynaecology and Obstetrics, University Hospital of Schleswig-Holstein, Campus Kiel, Christian-Albrechts University Kiel, Kiel, Germany; 5 Institute of Human Genetics, University Medical Center, Georg August University, Goettingen, Germany; 6 Institute of Human Genetics, University Medical Center Hamburg-Eppendorf, Hamburg, Germany; 7 Preventive Cardiology and Preventive Medicine, Center for Cardiology, University Medical Center of the Johannes Gutenberg-University Mainz, Mainz, Germany; 8 Cologne Center for Genomics, University of Cologne, Cologne, Germany; 9 Center for Molecular Medicine Cologne (CMMC), University of Cologne, Cologne, Germany; 10 Institute for Medical Informatics, Statistics and Epidemiology, University of Leipzig, Leipzig, Germany; 11 LIFE Leipzig Research Centre for Civilization Diseases, University of Leipzig, Leipzig, Germany; 12 Department of Gynaecology and Obstetrics, University of Munich, Campus Großhadern, Munich, Germany

**Keywords:** Early onset breast cancer, Ovarian cancer, *BARD1*, Germline mutations

## Abstract

**Background:**

The role of the *BARD1* gene in breast cancer (BC) and ovarian cancer (OC) predisposition remains elusive, as published case-control investigations have revealed controversial results. We aimed to assess the role of deleterious *BARD1* germline variants in BC/OC predisposition in a sample of 4920 *BRCA1/2*-negative female BC/OC index patients of the German Consortium for Hereditary Breast and Ovarian Cancer (GC-HBOC).

**Methods:**

A total of 4469 female index patients with BC, 451 index patients with OC, and 2767 geographically matched female control individuals were screened for loss-of-function (LoF) mutations and potentially damaging rare missense variants in *BARD1*. All patients met the inclusion criteria of the GC-HBOC for germline testing and reported at least one relative with BC or OC. Additional control datasets (Exome Aggregation Consortium, ExAC; Fabulous Ladies Over Seventy, FLOSSIES) were included for the calculation of odds ratios (ORs).

**Results:**

We identified LoF variants in 23 of 4469 BC index patients (0.51%) and in 36 of 37,265 control individuals (0.10%), resulting in an OR of 5.35 (95% confidence interval [CI] = 3.17–9.04; *P* < 0.00001). *BARD1-*mutated BC index patients showed a significantly younger mean age at first diagnosis (AAD; 42.3 years, range 24–60 years) compared with the overall study sample (48.6 years, range 17–92 years; *P* = 0.00347). In the subgroup of BC index patients with an AAD < 40 years, an OR of 12.04 (95% CI = 5.78–25.08; *P* < 0.00001) was observed. An OR of 7.43 (95% CI = 4.26–12.98; *P* < 0.00001) was observed when stratified for an AAD < 50 years. LoF variants in *BARD1* were not significantly associated with BC in the subgroup of index patients with an AAD ≥ 50 years (OR = 2.29; 95% CI = 0.82–6.45; *P* = 0.11217). Overall, rare and predicted damaging *BARD1* missense variants were significantly more prevalent in BC index patients compared with control individuals (OR = 2.15; 95% CI = 1.26–3.67; *P* = 0.00723). Neither LoF variants nor predicted damaging rare missense variants in *BARD1* were identified in 451 familial index patients with OC.

**Conclusions:**

Due to the significant association of germline LoF variants in *BARD1* with early-onset BC, we suggest that intensified BC surveillance programs should be offered to women carrying pathogenic *BARD1* gene variants.

**Electronic supplementary material:**

The online version of this article (10.1186/s13058-019-1137-9) contains supplementary material, which is available to authorized users.

## Background

The BRCA1-associated RING domain protein-1 (BARD1) was initially reported as a BRCA1-interacting protein by Wu et al. in 1996 [1]. The BRCA1 and BARD1 proteins show high structural homology, as they share *N*-terminal RING finger domains and BRCA1 C-terminal (BRCT) domains. Both proteins can form homodimers via their *N*-terminal RING finger domains [2, 3] but preferentially form more stable heterodimers involving amino acid residues 1–109 of the BRCA1 protein and amino acid residues 26–119 of the BARD1 protein [4]. The interaction between BARD1 and BRCA1 promotes tumor suppressor functions by acting in double-strand break repair and apoptosis initiation.

While the role of the *BRCA1* gene (MIM *113705) in breast cancer (BC) and ovarian cancer (OC) predisposition is well established [5], the role of *BARD1* (MIM *601593) in BC/OC predisposition remains elusive. Several case-control studies have investigated the association between deleterious germline variants in *BARD1* and the risk of developing female BC. Slavin et al. identified deleterious *BARD1* variants in 7 of 2134 *BRCA1/2*-negative familial BC patients (carrier frequency = 0.33%) and reported *BARD1* as a moderate-risk BC predisposition gene with an odds ratio (OR) of 3.18 (95% confidence interval [CI] = 1.34–7.36; *P* = 0.012) [6]. The considerably larger investigation of 28,536 BC patients of European ancestry by Couch et al. [7] revealed a carrier frequency of 0.18% (52/28,536) in BC patients and an OR of 2.16 (95% CI = 1.31–3.63; *P* = 0.00226). In contrast, however, recent studies by Lu et al. and Castéra et al. encompassing 9639 and 3667 patients with BC, respectively, did not show a significant association of deleterious *BARD1* variants with overall BC risk [8, 9]. Studies investigating the association of deleterious *BARD1* germline variants with OC risk, likewise, showed contradictory results. Norquist et al. identified protein-truncating germline variants in 4 of 1915 OC patients unselected for age or family history and in 18 of 36,276 control individuals, resulting in an OR of 4.2 (95% CI = 1.4–12.5; *P* = 0.02) [10]. In contrast, Ramus et al. were unable to demonstrate a significant association with OC in their study of 3261 unselected patients with epithelial OC and 3449 control individuals (4/3261, carrier frequency = 0.12%; 2/3449, carrier frequency = 0.06%; *P* = 0.39) [11]. Lilyquist et al. found deleterious *BARD1* germline variants in 8 of 6294 OC patients (carrier frequency = 0.13%) and calculated a nonsignificant risk ratio of 1.28 (95% CI = 0.55–2.51; *P* = 0.59) for OC [12]. Taken together, the role of deleterious *BARD1* germline variants in BC/OC predisposition remains unclear. In this study, we investigated the prevalence of deleterious *BARD1* germline variants in a sample of 4469 familial BC and 451 familial OC index patients of the German Consortium for Hereditary Breast and Ovarian Cancer (GC-HBOC) and 2767 geographically matched female controls (GMCs).

## Methods

### Study sample

A total of 4469 index patients with BC (mean age at first diagnosis [AAD] 48.6 years; range 17–92 years), 451 index patients with OC (mean AAD 53.4 years; range 18–85 years), and 2767 GMCs were screened for germline variants in *BARD1* (transcript NM_000465.3). All patients met the inclusion criteria of the GC-HBOC for germline testing [13] (Additional file 1: Table S1) and had at least one relative with BC or OC. Index patients with a personal history of both BC and OC were not included in this study. All patients were screened for pathogenic germline variants in BC/OC predisposition genes in a routine diagnostic setting using the TruRisk® gene panel of the GC-HBOC and tested negative for pathogenic *BRCA1/2* germline variants. Of the 4469 familial BC index patients, 3651 had a BC family history and no OC family history. Of the remaining 818 BC index patients, at least one family member with OC was reported. GMCs were aged 40 years and above and were cancer-free at the time of blood draw (mean age at blood draw 64.2 years; range 40–92 years). Written informed consent was obtained from all patients and controls; ethical approval was granted by the Ethics Committee of the University of Cologne (07-048). Two publicly accessible control datasets were used in this study (Table 1). From the Exome Aggregation Consortium (ExAC) [14], we requested a dataset of individuals of European, non-Finnish ancestry, excluding samples from The Cancer Genome Atlas (TCGA). This dataset comprises a total of 27,173 samples that were analyzed by whole-exome sequencing. The Fabulous Ladies Over Seventy (FLOSSIES) project provides a dataset of 7325 women of European American ancestry (https://whi.color.com). All participating women have remained cancer-free until at least 70 years of age. Blood-derived DNA samples of all participants were screened for variants in 27 established or suggested BC predisposition genes, including *BARD1*.

**Table 1:** Prevalence of heterozygous germline loss-of-function (LoF) variants identified in the *BARD1* gene (transcript NM_000465.3) in controls and index patients with breast cancer (BC) or ovarian cancer (OC) according to family history and age at first diagnosis (AAD). A total of 26 heterozygous germline LoF variants were listed in the ExAC database (Exome Aggregation Consortium, non-Finnish Europeans (NFE); excluding The Cancer Genome Atlas data (TCGA); as of June 2016); 8 heterozygous germline LoF variants were listed in the FLOSSIES database (“Fabulous Ladies Over Seventy”; American-European ancestry); 2 heterozygous germline LoF variants were identified in geographically matched female controls (GMCs); 23 germline LoF variants were found in 4469 familial index patients with BC; no heterozygous germline LoF variant was found in 451 familial index patients with OC. Univariate logistic regression was performed to estimate odds ratios (ORs) and 95% confidence intervals (CIs). When considering ExAC NFE nonTCGA controls only, ORs were similar to those given in Table 1 which consider all controls (^A^OR = 5.40, 95% CI = 3.08–9.47, *P* < 0.00001; ^B^OR = 5.46, 95% CI = 3.02–9.88, P < 0.00001; ^C^OR = 5.13, 95% CI = 1.79–14.74, *P* = 0.01084; ^D^OR = 12.16, 95% CI = 5.68–26.03, *P* < 0.00001; ^E^OR = 7.51, 95% CI = 4.15–13.58, *P* < 0.00001; ^F^OR = 5.58, 95% CI = 2.69–11.60, *P* = 0.00007; ^G^OR = 2.74, 95% CI = 0.83–9.08, *P* = 0.11082; ^H^OR = 2.32, 95% CI = 0.81–6.64, *P* = 0.11396, ^I^OR = 1.58, 95% CI = 0.21–11.66, *P* = 0.47806)

Study sample	Heterozygous carriers/number of tested individuals	Carrier frequency (%)	OR	95% CI	*P* value (Fisher’s exact test)
ExAC NFE nonTCGA	26/27,173	0.10	/	/	/
FLOSSIES	8/7325	0.11	/	/	/
GMCs	2/2767	0.07	/	/	/
All controls	36/37,265	0.10	/	/	/
Familial BC index patients	23/4469	0.51	5.35^A^	3.17–9.04	< 0.00001
Relative(s) with BC only	19/3651	0.52	5.41^B^	3.10–9.44	< 0.00001
Relative(s) with OC	4/818	0.49	5.08^C^	1.81–14.31	0.01046
AAD < 40	9/782	1.15	12.04^D^	5.78–25.08	< 0.00001
AAD < 50	19/2662	0.71	7.43^E^	4.26–12.98	< 0.00001
AAD 40–49	10/1880	0.53	5.53^F^	2.74–11.16	0.00005
AAD 50–59	3/1145	0.26	2.72^G^	0.84–8.83	0.10969
AAD ≥ 50	4/1807	0.22	2.29^H^	0.82–6.45	0.11217
AAD ≥ 60	1/662	0.15	1.57^I^	0.21–11.43	0.47891
Familial OC index patients	0/451	/	/	/	/
Relative(s) with BC only	0/379	/	/	/	/
Relative(s) with OC	0/72	/	/	/	/

### Next-generation sequencing (NGS)

Genomic DNA was isolated from venous blood samples. NGS and data analyses were carried out at each participating GC-HBOC center using Illumina sequencing platforms (MiSeq or NextSeq; Illumina, San Diego, CA, USA), employing the customized hybridization capture-based TruRisk® gene panel for target enrichment (manufactured by Agilent, Santa Clara, CA, USA; or Illumina). The diagnostic pipelines of the labs involved have been successfully tested in European Molecular Genetics Quality Network (EMQN) schemes. Predictions of large genomic rearrangements (LGRs) on the basis of NGS data are prone to give false-positive results and thus require validation. To date, no multiplex ligation-dependent probe amplification (MLPA) assay for the *BARD1* gene is commercially available. Thus, we did not include LGRs in our investigation [15].

### Variant classification

Variant classification was performed as previously described [16]. Briefly, all genetic variants were classified using a five-tier variant classification system as proposed by the International Agency for Research on Cancer (IARC) Unclassified Genetic Variants Working Group, namely, deleterious = class 5, likely deleterious = class 4, variant of uncertain significance (VUS) = class 3, likely benign = class 2, and benign = class 1. Variants reported to occur in large outbred control reference groups at an allele frequency of > 1% were generally considered benign. Loss-of-function (LoF) variants were defined as nonsense, frameshift, or essential splice site mutations affecting invariant splice sites or the last nucleotide of an exon. Missense variants were defined as potentially damaging when predicted deleterious by the in silico prediction tools SIFT and MutationTaster (Alamut version 2.10 as of November 9, 2017). Missense variants with a minor allele frequency (MAF) of < 0.1% in ExAC (non-Finnish Europeans; excluding TCGA data; as of June 2016) were defined as rare. All pathogenic (class 4/5) germline variants identified in patients and GMCs were confirmed by Sanger sequencing.

## Results

In our study sample of 4469 familial BC index patients, 23 patients carried heterozygous germline LoF variants in *BARD1*, resulting in a carrier frequency of 0.51% (Table 1). One *BARD1-*mutated BC index patient additionally carried a heterozygous germline LoF variant in the *CHEK2* gene (patient 5; c.902del, p.Glu301Glyfs*; Additional file 1: Table S2). The remaining 22 *BARD1-*mutated index patients tested negative for pathogenic variants in further BC/OC predisposition genes (*ATM*, *BRCA1*, *BRCA2*, *CDH1*, *CHEK2*, *PALB2*, *RAD51C*, *RAD51D*, and *TP53*). Information regarding the hormone receptor (estrogen receptor [ER]/progesterone receptor [PR]) and human epidermal growth factor receptor 2 (HER2) status of the tumor was available for 20/23 *BARD1-*mutated index patients with BC (Additional file 1: Table S2). Most *BARD1-*mutated index patients with BC developed hormone receptor-positive (ER-positive: 15/20; PR-positive: 11/20) and HER2-negative tumors (20/20). A triple-negative tumor phenotype was reported for 4 of 20 *BARD1-*mutated index patients with BC (Additional file 1: Table S2).

The carrier frequency observed in 4469 familial index patients with BC was elevated compared with the carrier frequencies observed in control datasets, which ranged from 0.07% (GMCs) to 0.11% (FLOSSIES) (Table 1). The comparison of carrier frequencies in the study sample of 4469 familial index patients with BC (23/4469, carrier frequency = 0.51%) and all control individuals (36/37,265, carrier frequency = 0.10%) revealed an OR of 5.35 (95% CI = 3.17–9.04; *P* < 0.00001) (Table 1). The subgroup of index patients with BC and heterozygous germline LoF variants in *BARD1* showed a younger mean AAD of BC (42.3 years; range 24–60 years) compared with the overall sample of index patients with BC (48.6 years; range 17–92 years), with differences reaching levels of significance (*P* = 0.00347; Student’s *t* test). When comparing LoF variant prevalence in the subgroup of index patients with BC and an AAD < 40 years and all control individuals, an OR of 12.04 (95% CI = 5.78–25.08; *P* < 0.00001) was observed (Table 1). An OR of 7.43 (95% CI = 4.26–12.98; *P* < 0.00001) was observed when stratified for an AAD < 50 years. Heterozygous germline LoF variants in *BARD1* were not significantly associated with BC in the subgroup of 1807 BC index patients with an AAD ≥ 50 years, although the ORs were marginally elevated (Table 1). All heterozygous germline LoF variants in *BARD1* identified in patients with BC and in control individuals are listed in the supplements (Additional file 1: Table S3).

Data on proven pathogenic *BARD1* missense variants are currently lacking [17–22]. To examine the potential association of missense variants in *BARD1* with BC risk, we focused on potentially damaging rare missense variants (MAF < 0.1%), which were predicted to be damaging by the SIFT and MutationTaster algorithms. The carrier frequency of potentially damaging rare *BARD1* missense variants was 0.18% for all control individuals (66/37,265; Additional file 1: Table S4). Rare *BARD1* missense variants predicted to be damaging by both tools were significantly more prevalent in index patients with BC compared with control individuals (17/4469, carrier frequency = 0.38%; OR = 2.15; 95% CI = 1.26–3.67; *P* = 0.00723; Additional file 1: Table S4). A slightly elevated association was observed for potentially damaging rare *BARD1* missense variants affecting the two BRCT domains spanning the amino acid residues 560–653 and 667–777 (9/4469, carrier frequency = 0.20%; OR = 2.42; 95% CI = 1.15–5.09; *P* = 0.03398; Additional file 1: Table S4).

In summary, *BARD1* appears to be a risk gene for early-onset familial BC. To avoid a recruitment bias by OC, we next stratified the study sample according to family history. In the subgroup of 3651 index patients with BC and without an OC family history (mean AAD 48.3 years; range 19–91 years), 19 patients carried heterozygous germline LoF variants in *BARD1*, resulting in a carrier frequency of 0.52% and an OR of 5.41 (95% CI = 3.10–9.44; *P* < 0.00001) compared with all control individuals (Table 1). In the subgroup of 818 index patients with BC and at least one relative with OC (mean AAD 50.1 years; range 17–92 years), 4 index patients carried heterozygous germline LoF variants in *BARD1* (carrier frequency = 0.49%) and an OR of 5.08 (95% CI = 1.81–14.31; *P* = 0.01046) compared with all control individuals. Thus, an OC family history did not affect the prevalence of *BARD1* LoF variants. The analysis of 451 familial index patients with OC (mean AAD 53.4 years; range 18–85 years) did not reveal heterozygous germline LoF variants in *BARD1* (Table 1), and none of the patients with OC carried potentially damaging rare *BARD1* missense variants.

## Discussion

We did not observe evidence that deleterious *BARD1* gene variants predispose for OC. LoF germline variants in *BARD1* could neither be detected in 451 familial OC index patients investigated in this study nor in our previously published analysis of 523 consecutive OC patients enrolled in the observational AGO-TR1 study [23]. Our data are in line with the data provided by Ramus et al. [11] and the largest investigation to date of 6294 OC cases by Lilyquist et al. [12], which showed a similar *BARD1* mutation prevalence in OC patients and controls. The weak association previously described by Norquist et al. (*P* = 0.02) [10] was based on the identification of 4 *BARD1-*mutated individuals in a study sample of 1915 unselected OC patients. Of note, Norquist et al. indicated that these results should be interpreted with some caution as 2 of the 4 *BARD1* mutation carriers also had mutations in *BRCA1* [10]. Overall, it appears likely that deleterious germline *BARD1* variants do not predispose for OC.

In study samples selected for (positive) cancer family history, the prevalence of deleterious variants in established risk genes is generally higher than in unselected cases. In our study focusing on 4469 index patients with familial BC, we demonstrate a significant association of heterozygous germline LoF variants in *BARD1* and overall BC (OR = 5.35; 95% CI = 3.17–9.04; *P* < 0.00001). This association is comparable with that described by Slavin et al. (OR = 3.18; 95% CI = 1.34–7.36; *P* = 0.012), a study that also focused on index cases with familial BC. In study samples unselected for family history, the observed ORs were lower (e.g., Couch et al.: OR = 2.16; 95% CI = 1.31–3.63; *P* = 0.00226) [7] and even nonsignificant (e.g., Castéra et al.: OR = 2.00; 95% CI = 0.74–4.10) [8, 9]. Thus, it appears worthwhile to stratify study results by family history and possibly AAD, as shown in the current study. We demonstrate a significant association of heterozygous germline LoF variants in *BARD1* and the risk of early-onset BC (Table 1), a finding which may have important implications for the clinical management of women carrying pathogenic *BARD1* variants. Due to the pronounced association with early-onset BC (AAD < 40 years: OR = 12.04; AAD < 50 years: OR = 7.43), we suggest that *BARD1* should be included in multigene panels for BC risk assessment and, due to the comparatively young AAD of BC observed, intensified BC surveillance programs should be offered to women carrying pathogenic variants in *BARD1*.

## Conclusions

No significant association between *BARD1* germline LoF variants and familial OC was observed. For BC, the significant association of heterozygous germline LoF variants in *BARD1* with early-onset BC (AAD < 50 years) suggests that intensified BC surveillance programs should be offered to women carrying pathogenic variants in *BARD1*.

## Additional file

Additional file 1: **Table S1.** Inclusion criteria of the German Consortium for Hereditary Breast and Ovarian Cancer (GC-HBOC) for *BRCA1* and *BRCA2* germline testing. **Table S2.** Genotype, phenotype and cancer family history of familial BC index patients carrying heterozygous germline loss-of-function (LoF) variants in the *BARD1* gene (transcript NM_000465.3). **Table S3.** Prevalence of heterozygous germline LoF variants identified in the *BARD1* gene (transcript NM_000465.3). **Table S4.** Potentially damaging rare missense variants identified in the *BARD1* gene (transcript NM_000465.3). (DOCX 76 kb)

## Abbreviations

AAD: Age at first diagnosis
BC: Breast cancer
CI: Confidence interval
EMQN: European Molecular Quality Network
ER: Estrogen receptor
ExAC: Exome Aggregation Consortium
FLOSSIES: Fabulous Ladies Over Seventy
GC-HBOC: German Consortium for Hereditary Breast and Ovarian Cancer
GMC: Geographically matched female control
HER2: Human epidermal growth receptor 2
IARC: International Agency for Research on Cancer
LGR: Large genomic rearrangement
LoF: Loss-of-function
MAF: Minor allele frequency
NGS: Next-generation sequencing
OC: Ovarian cancer
OR: Odds ratio
PR: Progesterone receptor
TCGA: The Cancer Genome Atlas
VUS: Variant of uncertain significance
