# Supplementary material for: Germline loss-of-function variants in the BARD1 gene are associated with early-onset familial breast cancer but not ovarian cancer
Source: Breast Cancer Res. 2019 Apr 29;21:55. doi: 10.1186/s13058-019-1137-9 (PMC6489184; doi:10.1186/s13058-019-1137-9)
Supplement: Supplementary file 1 — Table S1. Inclusion criteria of the German Consortium for Hereditary Breast and Ovarian Cancer (GC-HBOC) for BRCA1 and BRCA2 germline testing. Table S2. Genotype, phenotype and cancer family history of familial BC index patients carrying heterozygous germline loss-of-function (LoF) variants in the BARD1 gene (transcript NM_000465.3). Table S3. Prevalence of heterozygous germline LoF variants identified in the BARD1 gene (transcript NM_000465.3). Table S4. Potentially damaging rare missense variants identified in the BARD1 gene (transcript NM_000465.3). (DOCX 76 kb) [file 13058_2019_1137_MOESM1_ESM.docx]

**Table S1:** Inclusion criteria of the German Consortium for Hereditary Breast and Ovarian Cancer (GC-HBOC) for *BRCA1* and *BRCA2* germline testing. Ductal carcinoma *in situ* (DCIS) is categorized as breast cancer.

- ≥3 women with breast cancer
- ≥2 women with breast cancer, 1 with onset below 51 years of age
- ≥1 woman with breast cancer and 1 woman with ovarian cancer
- ≥2 women with ovarian cancer
- ≥1 woman with breast and ovarian cancer
- ≥1 woman with breast cancer below 36 years of age
- ≥1 woman with bilateral breast cancer with onset below 51 years
- ≥1 male with breast cancer and 1 woman with breast or ovarian cancer

**Table S2:** Genotype, phenotype and cancer family history of familial BC index patients carrying heterozygous germline loss-of-function (LoF) variants in the *BARD1* gene (transcript NM_000465.3). Patient #5 additionally carried a heterozygous germline LoF variant in the *CHEK2* gene (transcript NM_007194.3; c.902del, p.Glu301Glyfs*). All other *BARD1* LoF mutation carriers were negative for class 4/5 variants in other predisposition genes analyzed (*ATM, BRCA1, BRCA2, CDH1, CHEK2, PALB2, RAD51C, RAD51D,* and *TP53*). Given are the cancer sites and age at first diagnosis, family history (excluding index patients), tumor histology, grading, and tumor receptor status. ER = estrogen receptor, PR = progesterone receptor, HER2 = human epidermal growth factor receptor 2, TNBC = triple negative breast cancer, / = data not available.

| patient # | *BARD1* mutation (HGVS) | | cancer site  (age at diagnosis) | family history  (age at diagnosis) | tumor histology | grading | ER | PR | HER2 | TNBC |
| --- | --- | --- | --- | --- | --- | --- | --- | --- | --- | --- |
| BC index patients, with BC only family history | | | | | | | | | | |
| 1 | c.159-1G>T, p.? | BC(50) | | 2xBC(50,65) | non special type, DCIS | G2 | pos | pos | neg | no |
| 2 | c.159-2A>C, p.? | BC(39) | | 2xBC(46,80) | non special type, DCIS | G2 | neg | pos | neg | no |
| 3 | c.334C>T, (p.Arg112*) | BC(35) | | 1xBC(33) | non special type | G3 | neg | neg | neg | yes |
| 4 | c.334C>T, (p.Arg112*) | BC(40) | | 1xBC(35) | invasive lobular | G2 | pos | pos | neg | no |
| 5 | c.448C>T, (p.Arg150*) | BC(37) | | 1xBC(40) | non special type | G2 | pos | neg | neg | no |
| 6 | c.507delA, p.Asp172Metfs*40 | BC(49) | | 2xBC(32,40) | non special type | G3 | neg | neg | neg | yes |
| 7 | c.1216C>T, (p.Arg406*) | BC(53) | | 2xBC(40,85) | non special type | G3 | pos | neg | neg | no |
| 8 | c.1315-2A>G, p.? | BC(43) | | 1xBC(62) | non special type | G1 | pos | pos | neg | no |
| 9 | c.1396-1G>A, p.? | BCbil(45,45) | | 1xBC(>50) | invasive lobular, invasive lobular | G2, G3 | pos, pos | pos, pos | neg, neg | no, no |
| 10 | c.1690C>T, (p.Gln564*) | BC(24) | | 1xBC(43) | non special type | G3 | pos | pos | neg | no |
| 11 | c.1690C>T, (p.Gln564*) | BC(34) | | 1xBC(63) | / | / | / | / | / | / |
| 12 | c.1690C>T, (p.Gln564*) | BCbil(36,41) | | 1xBCbil(50,59) | medullary, non special type | G3, G3 | neg, neg | neg, neg | neg, neg | yes, yes |
| 13 | c.1690C>T, (p.Gln564*) | BC(42) | | 1xBC(58) | non special type | G2 | pos | neg | neg | no |
| 14 | c.1690C>T, (p.Gln564*) | BC(43) | | 1xBCbil (60,64) | invasive lobular | G2 | pos | pos | neg | no |
| 15 | c.1690C>T, (p.Gln564*) | BC(58) | | 1xBC(<51) | invasive lobular | G2 | pos | pos | neg | no |
| 16 | c.1771delA, p.Ile591Phefs*14 | BC(24) | | 1xBC(75) | unifocal, noninvasive DCIS | G3 | pos | pos | neg | no |
| 17 | c.1935_1954dup, p.Glu652Valfs*69 | BC(46) | | 5xBC(50,58,58,63,78) | invasive lobular | G2 | pos | pos | neg | no |
| 18 | c.2002-2A>C, p.? | BC(35) | | 2xBC(40,57) | squamous | / | / | / | / | / |
| 19 | c.2002-2A>C, p.? | BC(47) | | 2xBC(50,58) | non special type | G3 | pos | neg | neg | no |
| BC index patients, with OC family history | | | | | | | | | | |
| 20 | c.624delinsGG, p.Lys209Glufs*5 | BC(60) | | 1xOC(38) | DCIS | / | / | / | / | / |
| 21 | c.1216C>T, (p.Arg406*) | BC(36) | | 1xBC(48), 2xOC(45,75) | non special type, lobular | G3 | pos | pos | neg | no |
| 22 | c.1652C>G, (p.Ser551*) | BC(48) | | 3xBC(33,57,63), 1xOC(66) | non special type | G2 | pos | neg | neg | no |
| 23 | c.1690C>T, (p.Gln564*) | BC(49) | | 1xBC(50), 1xOC(40) | non special type | G2 | neg | neg | neg | yes |

**Table S3:** Prevalence of heterozygous germline LoF variants identified in the *BARD1* gene (transcript NM_000465.3). A total of 26 germline LoF variants were listed in the ExAC database (Exome Aggregation Consortium, non-Finnish Europeans; excluding The Cancer Genome Atlas data; as of June 2016); 8 germline LoF variants were listed in the FLOSSIES database (“Fabulous Ladies Over Seventy”, American-European ancestry) and 2 germline LoF variants were identified in geographically matched female controls (GMCs); 23 germline LoF variants were found in 4469 familial index patients with BC. All LoF variants identified in index patients and GMCs were verified by standard Sanger sequencing. Our analysis did not cover large genomic rearrangements (LGRs). LGRs reported in the ExAC/FLOSSIES databases were excluded. ^#^ = variant affects last nucleotide of an exon, / = variant not present in study sample, . = no rs-number.

| genomic position  (GRChr37/hg19) | cDNA position | protein | exon position | rs-number | ExAC (total) | ExAC  (%) | FLOSSIES (total) | FLOSSIES  (%) | GMCs  (total) | GMCs  (%) | familial BC index patients  (total) | familial BC index patients  (%) |
| --- | --- | --- | --- | --- | --- | --- | --- | --- | --- | --- | --- | --- |
| 2:215593409 | c.2324_2325del | p.Leu775Argfs*19 | 11/11 | rs587782046 | / | / | 1/7325 | 0.014 | / | / | / | / |
| 2:215593432 | c.2300_2301del | p.Val767Aspfs*4 | 11/11 | rs750413473 | 5/27,125 | 0.018 | 1/7325 | 0.014 | / | / | / | / |
| 2:215593535 | c.2199C>A | (p.Cys733*) | 11/11 | rs773179856 | 1/27,156 | 0.004 | / | / | / | / | / | / |
| 2:215593734 | c.2002-2A>C | p.? | 11/11 | rs876658260 | / | / | / | / | / | / | 2/4469 | 0.045 |
| 2:215595134 | c.2001+1G>T | p.? | 11/11 | rs768490891 | 1/27,144 | 0.004 | / | / | / | / | / | / |
| 2:215595181 | c.1935_1954dup | p.Glu652Valfs*69 | 10/11 | rs587780024 | / | / | / | / | / | / | 1/4469 | 0.022 |
| 2:215595215 | c.1921C>T | (p.Arg641*) | 10/11 | rs587781948 | 1/27,148 | 0.004 | 1/7325 | 0.014 | / | / | / | / |
| 2:215609874 | c.1819del | p.Val607Leufs*12 | 9/11 | rs774883556 | 1/26,665 | 0.004 | / | / | / | / | / | / |
| 2:215610485 | c.1771del | p.Ile591Phefs*14 | 8/11 | . | / | / | / | / | / | / | 1/4469 | 0.022 |
| 2:215610566 | c.1690C>T | (p.Gln564*) | 8/11 | rs587780021 | 4/27,172 | 0.015 | 1/7325 | 0.014 | 1/2767 | 0.036 | 7/4469 | 0.157 |
| 2:215617196 | c.1652C>G | (p.Ser551*) | 7/11 | rs587781707 | 1/27,147 | 0.004 | / | / | / | / | 1/4469 | 0.022 |
| 2:215632206 | c.1568T>C | p.(Val523Ala)^#^ | 6/11 | rs587780017 | 4/27,122 | 0.015 | 3/7325 | 0.041 | 1/2767 | 0.036 | / | / |
| 2:215632379 | c.1396-1G>A | p.? | 6/11 | . | / | / | / | / | / | / | 1/4469 | 0.022 |
| 2:215634038 | c.1315-2A>G | p.? | 5/11 | . | / | / | / | / | / | / | 1/4469 | 0.022 |
| 2:215645283 | c.1314+1G>A | p.? | 4/11 | rs753785671 | 1/26,772 | 0.004 | / | / | / | / | / | / |
| 2:215645382 | c.1216C>T | (p.Arg406*) | 4/11 | rs377153250 | 2/27,139 | 0.007 | / | / | / | / | 2/4469 | 0.045 |
| 2:215645974 | c.624delinsGG | p.Lys209Glufs*5 | 4/11 | . | / | / | / | / | / | / | 1/4469 | 0.022 |
| 2:215646072 | c.526C>T | (p.Gln176*) | 4/11 | rs776851287 | 1/27,124 | 0.004 | / | / | / | / | / | / |
| 2:215646084 | c.513dup | p.Asp172Argfs*10 | 4/11 | rs746325928 | 1/27,141 | 0.004 | / | / | / | / | / | / |
| 2:215646085 | c.513del | p.Asp172Metfs*40 | 4/11 | . | / | / | / | / | / | / | 1/4469 | 0.022 |
| 2:215646150 | c.448C>T | (p.Arg150*) | 4/11 | rs730881411 | 1/27,093 | 0.004 | / | / | / | / | 1/4469 | 0.022 |
| 2:215646203 | c.395del | p.Leu132Cysfs*27 | 4/11 | . | / | / | 1/7325 | 0.014 | / | / | / | / |
| 2:215657051 | c.334C>T | (p.Arg112*) | 3/11 | rs758972589 | / | / | / | / | / | / | 2/4469 | 0.045 |
| 2:215661785 | c.215G>A | p.(Ser72Asn)^#^ | 2/11 | rs769820924 | 1/27,154 | 0.004 | / | / | / | / | / | / |
| 2:215661842 | c.159-1G>T | p.? | 2/11 | rs879254139 | / | / | / | / | / | / | 1/4469 | 0.022 |
| 2:215661843 | c.159-2A>C | p.? | 2/11 | . | / | / | / | / | / | / | 1/4469 | 0.022 |
| 2:215674136 | c.158G>A | p.(Cys53Tyr)^#^ | 1/11 | rs747582517 | 1/24,828 | 0.004 | / | / | / | / | / | / |
| cumulative carrier frequency | | | | | **26/27,173** | **0.099** | **8/7325** | **0.111** | **2/2767** | **0.072** | **23/4469** | **0.512** |

**Table S4:** Potentially damaging rare missense variants identified in the *BARD1* gene (transcript NM_000465.3). A total of 52 potentially damaging rare missense variants were listed in the ExAC database (Exome Aggregation Consortium, non-Finnish Europeans; excluding The Cancer Genome Atlas data; as of June 2016); 11 potentially damaging rare missense variants were listed in the FLOSSIES database (“Fabulous Ladies Over Seventy”, American-European ancestry), and 3 potentially damaging rare missense variants were identified in geographically matched female controls (GMCs); 17 potentially damaging rare missense variants were found in 4469 familial index patients with BC. / = variant not present in study sample, . = no rs-number.

| genomic position  (GRChr37/hg19) | cDNA position | protein | exon position | protein | rs-number | ExAC  (total) | ExAC  (%) | FLOSSIES  (total) | FLOSSIES  (%) | GMCs  (total) | GMCs  (%) | all controls  (total) | all controls  (%) | familial BC index patients  (total) | familial BC index patients  (%) |
| --- | --- | --- | --- | --- | --- | --- | --- | --- | --- | --- | --- | --- | --- | --- | --- |
| 2:215593443 | c.2291T>C | p.Ile764Thr | 11/11 | p.Ile764Thr | rs587780030 | 1/27,130 | 0.004 | 1/7325 | 0.014 | / | / | 2/37,265 | 0.005 | 1/4469 | 0.022 |
| 2:215593455 | c.2279C>T | p.Ser760Leu | 11/11 | p.Ser760Leu | rs730881425 | 3/27,134 | 0.011 | / | / | / | / | 3/37,265 | 0.008 | / | / |
| 2:215593482 | c.2252G>A | p.Arg751Gln | 11/11 | p.Arg751Gln | rs587782246 | 6/27,152 | 0.022 | / | / | / | / | 6/37,265 | 0.016 | / | / |
| 2:215593518 | c.2216A>G | p.Tyr739Cys | 11/11 | p.Tyr739Cys | rs777013688 | 1/27,155 | 0.004 | / | / | / | / | 1/37,265 | 0.003 | / | / |
| 2:215593537 | c.2197T>C | p.Cys733Arg | 11/11 | p.Cys733Arg | rs977584514 | / | / | / | / | / | / | / | / | 1/4469 | 0.022 |
| 2:215593539 | c.2195T>G | p.Phe732Cys | 11/11 | p.Phe732Cys | rs760201905 | 1/27,157 | 0.004 | / | / | / | / | 1/37,265 | 0.003 | / | / |
| 2:215593545 | c.2189A>C | p.Gln730Pro | 11/11 | p.Gln730Pro | rs876658253 | / | / | / | / | / | / | / | / | 1/4469 | 0.022 |
| 2:215593549 | c.2185G>T | p.Asp729Tyr | 11/11 | p.Asp729Tyr | . | / | / | / | / | / | / | / | / | 1/4469 | 0.022 |
| 2:215593563 | c.2171C>T | p.Ala724Val | 11/11 | p.Ala724Val | rs587782662 | 5/27,156 | 0.018 | 1/7325 | 0.014 | / | / | 6/37,265 | 0.016 | 1/4469 | 0.022 |
| 2:215593597 | c.2137G>A | p.Val713Met | 11/11 | p.Val713Met | rs546077003 | 1/27,147 | 0.004 | / | / | / | / | 1/37,265 | 0.003 | 1/4469 | 0.022 |
| 2:215593611 | c.2123A>G | p.Lys708Arg | 11/11 | p.Lys708Arg | rs372160908 | 1/27,146 | 0.004 | 1/7325 | 0.014 | / | / | 2/37,265 | 0.005 | / | / |
| 2:215593707 | c.2027A>C | p.Tyr676Ser | 11/11 | p.Tyr676Ser | rs770917842 | 1/27,037 | 0.004 | / | / | / | / | 1/37,265 | 0.003 | / | / |
| 2:215595151 | c.1985T>G | p.Leu662Arg | 10/11 | p.Leu662Arg | rs556775078 | / | / | 1/7325 | 0.014 | / | / | 1/37,265 | 0.003 | / | / |
| 2:215595221 | c.1915T>C | p.Cys639Arg | 10/11 | p.Cys639Arg | rs587781376 | 2/27,151 | 0.007 | 1/7325 | 0.014 | / | / | 3/37,265 | 0.008 | 2/4469 | 0.045 |
| 2:215609808 | c.1886G>T | p.Trp629Leu | 9/11 | p.Trp629Leu | rs747446711 | 1/26,783 | 0.004 | / | / | / | / | 1/37,265 | 0.003 | / | / |
| 2:215609826 | c.1868G>A | p.Gly623Glu | 9/11 | p.Gly623Glu | rs587782252 | 2/26,939 | 0.007 | / | / | / | / | 2/37,265 | 0.005 | / | / |
| 2:215610538 | c.1718T>C | p.Ile573Thr | 8/11 | p.Ile573Thr | rs587780022 | 2/27,172 | 0.007 | / | / | / | / | 2/37,265 | 0.005 | 1/4469 | 0.022 |
| 2:215617226 | c.1622C>T | p.Ser541Leu | 7/11 | p.Ser541Leu | rs777937955 | 1/27,152 | 0.004 | / | / | / | / | 1/37,265 | 0.003 | / | / |
| 2:215617263 | c.1585C>T | p.Arg529Trp | 7/11 | p.Arg529Trp | rs375515606 | 2/27,141 | 0.007 | / | / | 1/2767 | 0.036 | 3/37,265 | 0.008 | / | / |
| 2:215617277 | c.1571A>G | p.Asn524Ser | 7/11 | p.Asn524Ser | rs587781887 | 1/27,131 | 0.004 | / | / | / | / | 1/37,265 | 0.003 | / | / |
| 2:215632276 | c.1498G>A | p.Asp500Asn | 6/11 | p.Asp500Asn | rs779468443 | 1/27,140 | 0.004 | 1/7325 | 0.014 | / | / | 2/37,265 | 0.005 | / | / |
| 2:215632377 | c.1397A>T | p.His466Leu | 6/11 | p.His466Leu | . | / | / | / | / | / | / | / | / | 1/4469 | 0.022 |
| 2:215633991 | c.1360C>G | p.Pro454Ala | 5/11 | p.Pro454Ala | rs730881408 | 2/27,097 | 0.007 | / | / | 1/2767 | 0.036 | 3/37,265 | 0.008 | / | / |
| 2:215633999 | c.1352G>C | p.Gly451Ala | 5/11 | p.Gly451Ala | rs771410310 | 1/27,094 | 0.004 | / | / | / | / | 1/37,265 | 0.003 | / | / |
| 2:215634012 | c.1339C>G | p.Leu447Val | 5/11 | p.Leu447Val | rs376727038 | 4/27,078 | 0.015 | 1/7325 | 0.014 | / | / | 5/37,265 | 0.014 | 1/4469 | 0.022 |
| 2:215645286 | c.1312A>G | p.Lys438Glu | 4/11 | p.Lys438Glu | rs754539312 | 1/26,846 | 0.004 | / | / | / | / | 1/37,265 | 0.003 | / | / |
| 2:215645291 | c.1307C>G | p.Ser436Cys | 4/11 | p.Ser436Cys | rs752234478 | 1/26,926 | 0.004 | / | / | / | / | 1/37,265 | 0.003 | / | / |
| 2:215645298 | c.1300A>T | p.Ile434Phe | 4/11 | p.Ile434Phe | rs758049210 | / | / | / | / | 1/2767 | 0.036 | 1/37,265 | 0.003 | 1/4469 | 0.022 |
| 2:215645309 | c.1289C>G | p.Thr430Ser | 4/11 | p.Thr430Ser | rs587780015 | 2/27,053 | 0.007 | / | / | / | / | 2/37,265 | 0.005 | / | / |
| 2:215646059 | c.539A>G | p.Tyr180Cys | 4/11 | p.Tyr180Cys | . | / | / | / | / | / | / | / | / | 1/4469 | 0.022 |
| 2:215646141 | c.457A>G | p.Lys153Glu | 4/11 | p.Lys153Glu | rs753377280 | 1/27,102 | 0.004 | / | / | / | / | 1/37,265 | 0.003 | / | / |
| 2:215657092 | c.293A>C | p.Asn98Thr | 3/11 | p.Asn98Thr | . | / | / | / | / | / | / | / | / | 1/4469 | 0.022 |
| 2:215657096 | c.289A>G | p.Ile97Val | 3/11 | p.Ile97Val | . | / | / | / | / | / | / | / | / | 1/4469 | 0.022 |
| 2:215657104 | c.281A>C | p.Asp94Ala | 3/11 | p.Asp94Ala | rs757569689 | 1/27,084 | 0.004 | / | / | / | / | 1/37,265 | 0.003 | / | / |
| 2:215657119 | c.266C>T | p.Pro89Leu | 3/11 | p.Pro89Leu | rs780241203 | 1/27,057 | 0.004 | / | / | / | / | 1/37,265 | 0.003 | / | / |
| 2:215657164 | c.221G>C | p.Cys74Ser | 3/11 | p.Cys74Ser | . | / | / | 1/7325 | 0.014 | / | / | 1/37,265 | 0.003 | 1/4469 | 0.022 |
| 2:215661788 | c.212G>C | p.Cys71Ser | 2/11 | p.Cys71Ser | . | / | / | / | / | / | / | / | / | 1/4469 | 0.022 |
| 2:215661812 | c.188T>C | p.Leu63Ser | 2/11 | p.Leu63Ser | rs748828467 | 1/27,161 | 0.004 | / | / | / | / | 1/37,265 | 0.003 | / | / |
| 2:215674142 | c.152C>T | p.Ser51Leu | 1/11 | p.Ser51Leu | rs766788652 | 1/24,851 | 0.004 | / | / | / | / | 1/37,265 | 0.003 | / | / |
| 2:215674175 | c.119C>T | p.Ala40Val | 1/11 | p.Ala40Val | rs71579841 | 3/24,063 | 0.012 | 1/7325 | 0.014 | / | / | 4/37,265 | 0.011 | / | / |
| 2:215674217 | c.77T>G | p.Met26Arg | 1/11 | p.Met26Arg | rs759957629 | / | / | 1/7325 | 0.014 | / | / | 1/37,265 | 0.003 | / | / |
| 2:215674240 | c.54C>G | p.Asn18Lys | 1/11 | p.Asn18Lys | rs587780032 | / | / | 1/7325 | 0.014 | / | / | 1/37,265 | 0.003 | / | / |
| 2:215674245 | c.49G>A | p.Gly17Arg | 1/11 | p.Gly17Arg | rs746495820 | 1/10,543 | 0.009 | / | / | / | / | 1/37,265 | 0.003 | / | / |
| cumulative carrier frequency | | | | | | **52/27,173** | **0.201** | **11/7325** | **0.154** | **3/2767** | **0.108** | **66/37,265** | **0.182** | **17/4469** | **0.375** |
